# Vibration Detection and Motion Compensation for Multi-Frequency Phase-Shifting-Based 3D Sensors

**DOI:** 10.3390/s19061368

**Published:** 2019-03-19

**Authors:** Liya Han, Zhongwei Li, Kai Zhong, Xu Cheng, Hua Luo, Gang Liu, Junyun Shang, Congjun Wang, Yusheng Shi

**Affiliations:** 1 State Key Laboratory of Material Processing and Die & Mould Technology, Huazhong University of Science and Technology, Wuhan 430074, China; hly1993@hust.edu.cn (L.H.); xu_cheng@hust.edu.cn (X.C.); walden@263.net (C.W.); shiyusheng@hust.edu.cn (Y.S.); 2 Xi’an Aerospace Precision Electromechanical Institute, Xi’an 710100, China; louheather@163.com (H.L.); wuliusheng1994@foxmail.com (G.L.); wulanghust@gmail.com (J.S.); 3 Wuhan Vision 3D Technology Ltd., Wuhan 430074, China

**Keywords:** multi-frequency phase-shifting method, vibration detection, motion compensation

## Abstract

Phase-shifting profilometry, especially employing the multi-frequency phase-shifting method, is increasingly used for in situ 3D metrology and for the inspection of industrial parts. However, environmental vibrations cause fatal measurement errors and are inevitable in such applications. To this end, an effective and fast vibration detection and motion compensation method for multi-frequency phase-shifting-based 3D sensors is presented. The proposed method quantitatively indicates the strength of the vibration and compensates for the motion error by revising the wrapped phase without accessing neighboring pixels. Different vibration intensities were simulated using an industrial robot moving to test the feasibility of the method. According to experiments, this method is valid and capable for 3D inspection systems affected by inevitable vibrations.

## 1. Introduction

Industrial in situ 3D inspection is an optical inspection applied just after products have been manufactured in a production line [1]. Vibration is inevitable and sporadic in factories, especially forging factories, and may cause fatal errors to the optical systems. For an inspection system using phase-shifting profilometry (PSP), vibration may cause mistakes or losses in the reconstructed 3D data [2], which will affect the inspection results and confuse the following procedures. This kind of vibration is hard to prevent or predict, and the only economical and feasible solution is to detect if a vibration occurs and then try to compensate the motion error caused by the vibration.

Physical vibration sensors are actually acceleration sensors [3], which do not measure the vibration itself, but indicate the vibration intensity by the value of the vibration-caused acceleration. For typical sinusoidal vibration signals, the acceleration is proportional to the amplitude and square of the frequency. When the vibration frequency is relatively low, the acceleration will be below the detection criteria and therefore undetectable. Environmental vibrations in a forging factory are usually relatively low frequency due to the propagation characteristics of impact vibrations [4]. They affect the inspection system, but are hard to detect using physical sensors. Therefore, an image-based vibration detection method for phase-shifting profilometry is urgently needed in such situations.

Traditionally, the detection of vibrations in images utilizes the metric of image blur. Many solutions for natural images have been developed over the years [5], but these metrics cannot be easily applied to fringe projection images because of the grayscale edges artificially created by non-uniform illumination. For fringe pattern vibration detection, studies are more often reported in the field of interferometry fringe analysis [6] and are not suitable for the projected fringe pattern and incoherent light source in PSP.

PSP works under the principle of stereo vision. By projecting a series of fringe patterns onto an object to be scanned, corresponding points in two cameras can be located using the phase constraint and epipolar constraint [7]. For each camera, the phase information of the captured image is recovered from gray values of the same pixel in different frames [8]. From a sequence of phase-shifting images, a wrapped phase map can be established. If multiple sequences of phase-shifting images with different fringe frequencies are captured, the wrapped phase can be unwrapped with a multi-frequency heterodyne. This is achieved despite discontinuities on the surface since the absolute phase is unwrapped from the wrapped phase values of the same pixel in different wrapped phase maps [9]. The phase recovery and unwrapping are thus conducted pixel by pixel without accessing any neighborhood pixels [10]. The isolation of pixels helps overcome the unevenness, texture, and discontinuity of the object’s surface, which makes the multi-frequency phase-shifting method more accurate and stable than other methods that use fewer patterns, but also leads to greater sensitivity to vibrations.

Some work has been undertaken in recent years to compensate for the motion error of the phase map in PSP. Weise et al. proposed a method to estimate motion errors with a Taylor series [11]. With an assumption of local smoothness, they exploited a least-square fitting over a small neighborhood to estimate the motion-induced phase offset for each pixel. Cong et al. proposed a Fourier-assisted PSP approach, which corrected the phase shift error by differentiating the phase maps of two successive fringe images [12]. In addition, Lu et al. suggested refining the unknown phase shifts using the least-squares method with constraints of the background intensity and the modulation amplitude [13]. Feng et al. divided motion errors into three categories: motion ripples, motion-induced phase unwrapping errors, and motion outliers [14]. Different methods were applied in the three situations. The purpose of all of these methods was to perform high-speed 3D reconstruction of moving objects, where the speed requirements took precedence over accuracy. In these approaches, only the motion compensation of the phase-shifting images was considered, and the isolation of pixels was usually abandoned to estimate the phase error.

In most motion compensation studies, there is a basic assumption that the motion during the scanning period can be regarded as linear. Under this assumption, in a multi-frequency phase-shifting sequence composed of several subsequences of phase-shifting frames, the motion between frequencies is several times larger than the motion between the phase-shifting frames. In other words, motion errors between frequencies are more critical, relative to that within the phase-shifting subsequences. From motion compensation studies, it can be concluded that with a temporal phase-recovery algorithm, the fringe phases are only intermediate values that have a relatively large allowance for errors if the vibration or motion intensity is low, relative to the fringe cycle. In cases of relatively low frequency vibrations, the motion error within the phase-shifting subsequence of a certain frequency is small enough to be omitted, but the pixel correspondence between the frequencies may have been damaged and will lead to the failure of phase unwrapping. However, there have been few studies on the motion compensation of a multi-frequency phase-shifting sequence.

In this work, we present an image-based metric for the vibration intensity in a multi-frequency phase-shifting sequence and a novel motion compensation method based on the correction of pixel correspondence in which the isolation of pixels is retained. As mentioned above, a multi-frequency phase-shifting sequence is composed using subsequences of phase-shifting frames. Each subsequence generates a wrapped phase map and several wrapped phase maps generate the unwrapped phase map. 3D reconstruction failure is due to the error correspondence between wrapped phase maps, which is also the error correspondence between subsequences. In our method, the magnitude of the corresponding error between subsequences was measured and used to indicate the strength of the vibration. Furthermore, by correcting the correspondence between the wrapped phase maps, the motion error was compensated for and the correct phase unwrapping results were obtained. A 3D sensor operating under a four-step, three-frequency phase-shifting method was utilized in the implementation of this method. Therefore, this study is based on, but not limited to four-step, three-frequency fringe projection. To simplify the presentation, some of the figures used the two-frequency, three-step fringe projection to illustrate the principle, which is the simplest case of multi-frequency phase-shifting methods.

In order to verify the proposed method, a multi-frequency phase-shifting 3D sensor was fixed on the end effector of an industrial robot. Robot motion with different parameters was used to simulate vibrations of different intensities, and different targets were measured during this process to produce data that is affected by vibration. Vibration detection worked well under all parameters and targets, and motion compensation achieved good results below a certain vibration intensity. The factors limiting the effect of compensation were also summarized.

The chapters in this article are organized as follow: In Section 2, we study how vibration affects a multi-frequency phase-shifting sequence and, on this basis, explained the principles of vibration detection and motion compensation. In Section 3, we describe the purpose and specific implementation of the experiment. In Section 4, we provide the experimental results and analyze them. In the last section, we summarize the article and present our conclusions.

## 2. Materials and Methods

### 2.1. Vibration Effects on a Multi-Frequency Phase-Shifting Sequence

The basic components of a multi-frequency, phase-shifting 3D sensor are one projector and two cameras on either side. For each camera, the measurement process is the same, the essence of which is to continuously acquire a sequence of images. The cameras on both sides generate their own unwrapped phase maps, and then generate 3D point cloud data together based on the binocular vision principle. The principle analysis of the vibration effects only needs to be performed on one camera.

For one camera, when a sequence of images is affected by vibrations, the space constraints within the sequence are destroyed. By analyzing the structure of the sequence, we can find out how the sequence is affected and then try to indicate and compensate for the effects of vibrations.

In phase-shifting methods, a series of sinusoidal fringes along the horizontal axis of the projector image frame, with a constant phase shift, is projected onto a target object and two cameras synchronously capture the phase-encoded fringe images [15]. In particular, the captured images of the cameras can be expressed as:

(1) $$I_{ji}\left(x,y\right)=A\left(x,y\right)+B\left(x,y\right)\cos\left[\varphi_{j}\left(x,y\right)+\left(i-1\right)\delta \right]$$

where (x,y) denotes the pixel coordinates, which will be omitted in the following expressions; $I_{ji}$ is the recorded intensity of the ith frame at the jth frequency; A is the average intensity; B is the modulation intensity; δ is the constant phase shift; and $\varphi_{j}\left(x,y\right)$ is the desired phase information of the jth frequency. If N is the number of frames within a single frequency, we have:

(2) $$\varphi_{j}\left(x,y\right)=-\arctan\left(\sum_{i=1}^{N}I_{ji}\left(x,y\right)\sin\left(i-1\right)\delta /\sum_{i=1}^{N}I_{ji}\left(x,y\right)\cos\left(i-1\right)\delta \right)$$

**Figure 1:**
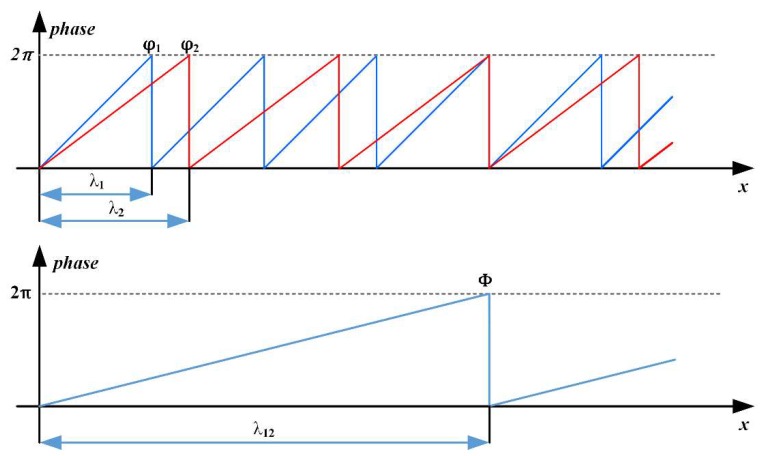
Heterodyne principle. $\varphi_{1}$ and $\varphi_{2}$ are wrapped phase functions and $\lambda_{1}$, $\lambda_{2}$ are corresponding wavelengths, respectively. Φ is the unwrapped phase function.

The calculation of $\varphi_{j}\left(x,y\right)$ is called phase recovery. However, $\varphi_{j}\left(x,y\right)$ is wrapped due to the periodicity of trigonometric functions. In order to unwrap $\varphi_{j}\left(x,y\right)$ to an absolute phase Φ(x,y), the value of $\varphi_{j}\left(x,y\right)$ for different frequencies j are needed to perform a heterodyne [9], which means using a combination of multiple frequencies to produce a frequency lower than any of these frequencies. Considering the unidirectionality of the fringe, $\varphi_{j}\left(x,y\right)$ and Φ(x,y) can be simplified to $\varphi_{j}\left(x\right)$ and Φ(x), respectively. As shown in Figure 1, the frequencies of the phase functions $\varphi_{j}\left(x\right)$ have to be chosen in a way that the resulting beat function Φ(x) is unambiguous over the field of view. For the situation of j={1,2}, $\lambda_{1}$ and $\lambda_{2}$ are corresponding wavelengths of $\varphi_{1}\left(x\right)$ and $\varphi_{2}\left(x\right)$, respectively, and the heterodyne wavelength $\lambda_{12}$ can be solved according to the following equation:

(3) $$\lambda_{12}=\frac{\lambda_{1}\lambda_{2}}{\lambda_{2}-\lambda_{1}}$$

If $\Phi_{1}\left(x\right)$ and $\Phi_{2}\left(x\right)$ are the unwrapped phases of $\varphi_{1}\left(x\right)$ and $\varphi_{2}\left(x\right)$, respectively, it is easy to get:

(4) $$\frac{\Phi_{1}\left(x\right)\lambda_{1}}{2\pi }=\frac{\Phi_{2}\left(x\right)\lambda_{2}}{2\pi }$$

Then, we have:

(5) $$\Delta \Phi \left(x\right)=\Phi_{1}\left(x\right)-\Phi_{2}\left(x\right)=\{\begin{array}{c} \varphi_{1}\left(x\right)-\varphi_{2}\left(x\right)\left(\varphi_{1}\geq \varphi_{2}\right)\\ 2\pi +\varphi_{1}\left(x\right)-\varphi_{2}\left(x\right)\left(\varphi_{1}<\varphi_{2}\right) \end{array}$$

From Equations (4) and (5), we have:

(6) $$\Phi_{1}\left(x\right)=\frac{\lambda_{2}}{\lambda_{2}-\lambda_{1}}\Delta \Phi \left(x\right)$$

or

(7) $$\{\begin{array}{c} m=round\left[\left(\frac{\lambda_{2}}{\lambda_{2}-\lambda_{1}}\Delta \Phi \left(x\right)-\varphi_{1}\left(x\right)\right)/2\pi \right]\\ \Phi_{1}\left(x\right)=2\pi m+\varphi_{1}\left(x\right) \end{array}$$

in which round is the rounding function. Therefore, we get a lower frequency $\Phi_{1}$ through $\varphi_{1}$ and $\varphi_{2}$. Similarly, we can continue the multi-frequency heterodyne if we have more $\varphi_{j}$ until the final Φ has only one cycle in the entire field of view.

To do so, we can divide the multi-frequency phase-shifting method into two parts: the phase recovery and the multi-frequency heterodyne. Both depend on a pixel correspondence between the frames, which means that the same pixel coordinates (x,y) in different frames from the same camera must represent the same point on the object. In the quiescent state, the frames are completely coincident in space, and the scenes contained therein are completely identical, but when a sequence is affected by a vibration, the coincidence between the frames is destroyed, and there is a certain degree of displacement between them.

**Figure 2:**
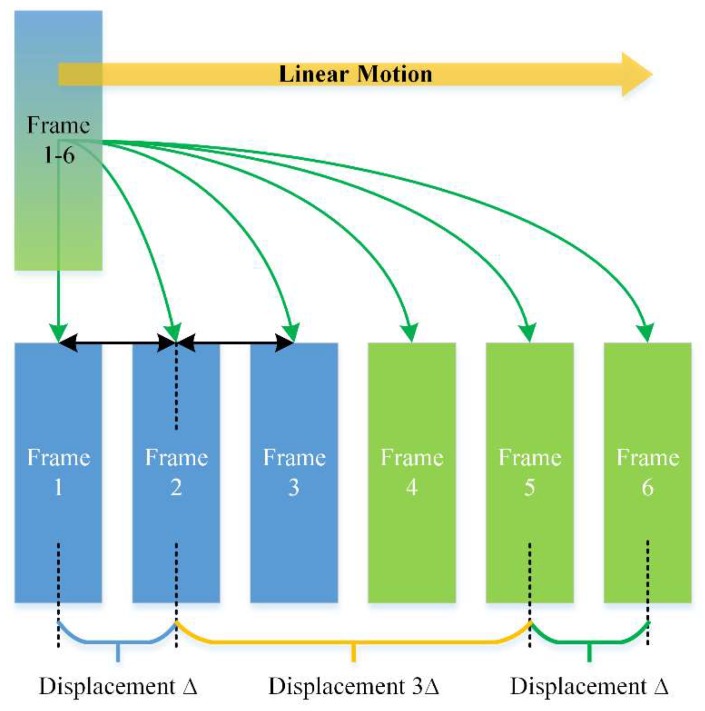
Displacements within a two-frequency, three-step phase-shifting sequence.

In a multi-frequency, phase-shifting sequence of *m* frequencies with *n* phases for each frequency, the motion between the frequencies can be regarded as the motion between the *i*th frame of one frequency and the *i*th frame of the neighboring frequency. Likewise, the motion between phases can be regarded as the motion between a frame and its neighboring frame within the same frequency. It is easy to understand that the displacement between frequencies is *n* times that between the phase-shifting frames. For example, in a two-frequency, three-step phase-shifting sequence affected by linear motion, there are six frames (Figure 2). Frames 1–3 belong to the first frequency and frames 4–6 belong to the second. The displacement within a frequency is ∆. Consider the second frame as the reference, then the displacement between the two frequencies is 3∆, which is three times that of the three-step phase-shifting subsequence. Based on this analysis, it can be considered that the multi-frequency heterodyne process is more susceptible to vibration than the phase recovery process.

**Figure 3:**
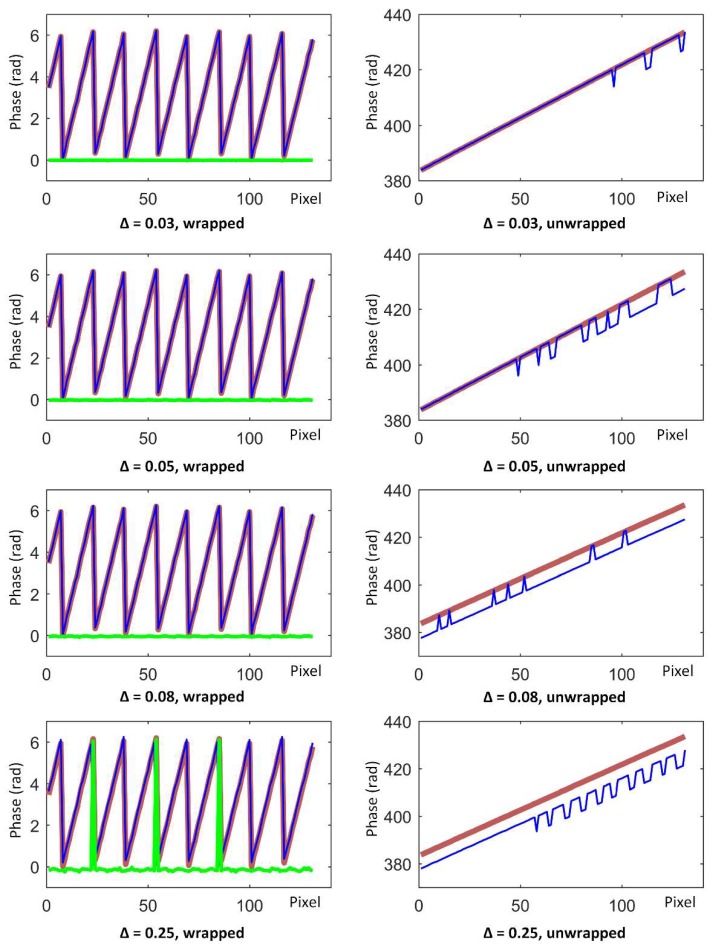
Phase value curves of the wrapped and unwrapped phase images with different ∆s. ∆ is the pixel displacement of frames. The thick red lines are phase curves of the original sequence, the thin blue lines are phase curves of the moved sequences, and the green lines are the phase errors.

To quantitatively clarify this concept, we designed the following experiment: we considered a three-frequency, four-step phase-shifting image sequence of a piece of paper, where the purpose of selecting a piece of paper as a target was to obtain a relatively linear unwrapped phase for easy comparison. We project 12 images of a three-frequency, four-step phase-shifting sequence onto a flat white paper and captured them synchronously with the camera. We moved each image ∆ pixels to the right, relative to the previous frame in a direction perpendicular to the fringes, which means that the *i*th image will move (*i* − 1)∆ pixels from its origin. Figure 3 shows the wrapped and unwrapped phases (obtained using the aforementioned methods) with different ∆s, before and after movement. The thick red lines are from the original sequence, the thin blue lines are from the moved sequence, and the green line is the phase error. In each graph, the abscissa is a pixel interval with a width of 140 pixels (unit: 1), and the ordinate is a phase value (unit: rad). From the experiment, we easily found that as ∆ rose from 0.03 to 0.08, the unwrapped phase error kept growing but the wrapped phase error was close to 0 and had no significant change. Until ∆ reached the considerable amount of 0.25, the wrapped phase error was easy to identify while the unwrapped phase error became more significant. This result means that in the process of gradually increasing the vibration intensity, the multi-frequency heterodyne process was affected first, when compared to the phase recovery process. In other words, below a certain vibration intensity, the phase-shifting subsequence was unaffected, but the constraints between multiple frequencies were destroyed, which is exactly the case of the relatively low frequency vibration discussed in this paper.

**Figure 4:**
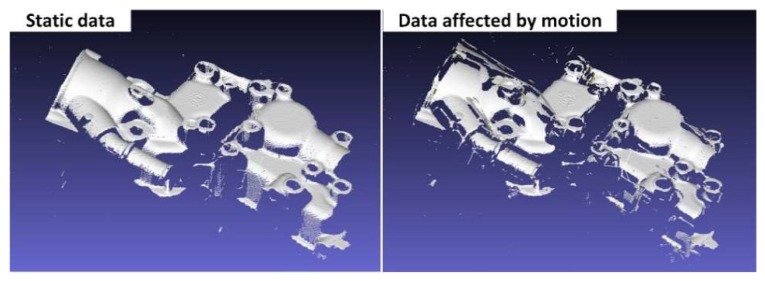
The “broken” surfaces in the 3D reconstruction caused by a destroyed pixel correspondence.

In the case of relatively low frequency vibrations, the motion error within the phase-shifting sequence of a certain frequency is small enough to be omitted or is easily removed using temporal phase-recovery algorithms, but the pixel correspondence between frequencies might be damaged. In these situations, there will be wrong phase unwrapping results and destroyed 3D reconstructions. As Figure 4 shows, the 3D reconstruction from a multi-frequency, phase-shifting sequence in motion will have “broken” surfaces, which is the main form of motion error in a multi-frequency phase-shifting sequence. As seen in Figure 3 when ∆ =0.03 and ∆ =0.05, the 3D data affected by the vibration may be intact in the localized region, but have a “broken” surface globally. This is different from the vibration-affected phase-shifting subsequence, where there will be global ripples, outliers, etc.

### 2.2. Vibration Detection and Motion Compensation

In the multi-frequency phase-shifting method, the information is redundant if the ambient light image, *A*, can be regarded as a constant [16]. For the *N*-step phase-shifting pattern sequence, knowing that δ=2π/N, we can easily obtain:

(8) $$\sum_{i=1}^{N}I_{ji}=N\cdot A$$

which means that the linear superposition of the fringe images will eliminate the streak component. If multiple reflections are ignored, the resulting image is no different than a uniformly illuminated image. In fact, in the measurement of non-high-reflecting objects, the superposition image is very close to the uniformly illuminated image; the absolute difference is almost negligible. In a multi-frequency phase-shifting sequence, the superposition of each frequency results in a uniformly illuminated image (which is known as a virtual frame) and the whole sequence can be fused as a series of uniformly illuminated virtual frames.

According to phase-shifting motion compensation studies, if the image sequence is affected by vibration or motion, an additional phase shift is introduced [11]. Under these circumstances, the linear superposition will still have the streak component. With an additional phase shift ω between frames and defining $P=\sum_{i=2}^{N}i\cos\left(i-1\right)\delta$ and $Q=\sum_{i=2}^{N}i\sin\left(i-1\right)\delta$, we have:

(9) $$\sum_{i=1}^{N}I_{ji}=N\cdot A-\omega B\sqrt{P^{2}+Q^{2}}sin\left[\varphi_{j}+\arctan\frac{Q}{P}\right]$$

**Figure 5:**
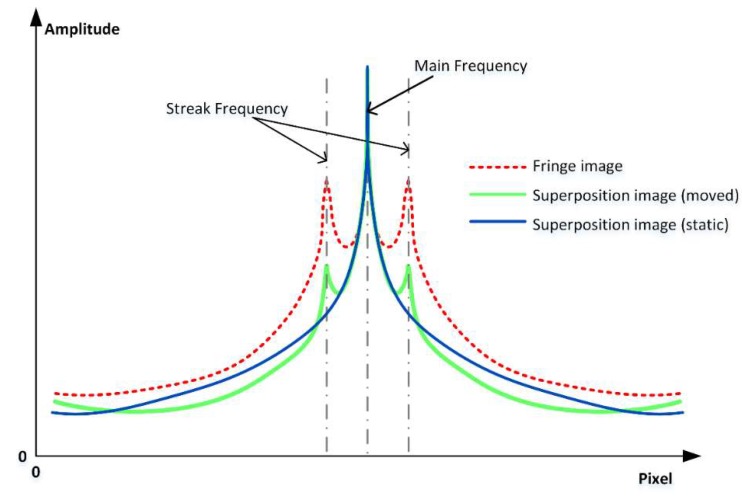
Schematic diagram of a streak frequency check based on Fourier transform maps. The red dotted line is from the fringe image, the blue line is from superposition image of static subsequence, and the green line is from superposition image of moved subsequence.

Obviously, the magnitude of the additional phase shift determines the strength of the streak component. In other words, the streak intensity indicates the magnitude of the vibration or motion. By extracting the ROI (region of interest) from the Fourier transform map of the fringe image and applying it to the superposition image, we can extract the peak of the streak frequency in the superposition image and compare it with the corresponding peak in the fringe image, as Figure 4 shows. It should be noted that Figure 5 is only a schematic diagram drawn according to the Fourier transform maps, and the data in the graph is not strictly accurate. There will be visible streaks if the vibration or motion is strong enough, but in relatively low frequency vibration situations, the additional phase shift ω may be too small for detection. In these situations, another criterion is proposed to quantitatively evaluate the motion intensity.

If the streak intensity is lower than a certain threshold, the streak component of the virtual frame can be omitted, and the grayscale feature point detection can be applied to the virtual frame. As mentioned in Section 2.1, in a motion or vibration situation, the same point on the object will have different pixel coordinates in neighboring frames. The difference between the feature point arrays of a virtual frame pair indicates the motion intensity between the two virtual frames. The L1-norm can be used as an indicator for this difference, which is positively correlated with vibration intensity. Supposing that $\vec{n_{1}}$ represents the feature point array in the first virtual frame and $\vec{n_{2}}$ in the second, and $N_{p}$ represents the number of points in $\vec{n_{1}}$ (which is also the number of points in $\vec{n_{2}}$), we have a metric $N_{L1}$ for the relative movement between the virtual frames:

(10) $$N_{L1}=\frac{\sum_{I}\left|\vec{n_{1}}\left(I\right)-\vec{n_{2}}\left(I\right)\right|}{N_{p}}$$

**Figure 6:**
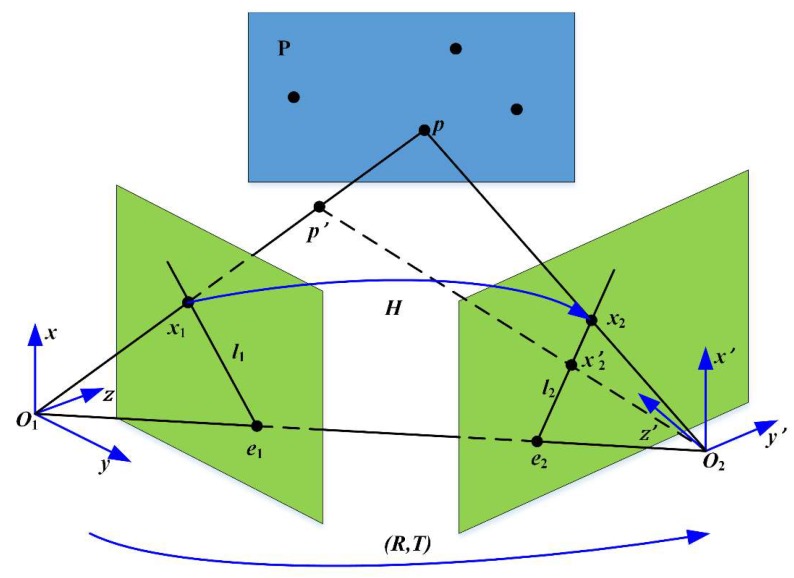
Parallax and homography. P is a plane. p is a point on plane P and $p^{\prime }$ is a point out of plane P. $O_{1}$ and $O_{2}$ are the optical center positions in two frames. $O_{1}-xyz$ and $O_{2}-x^{\prime }y^{\prime }z^{\prime }$ are camera coordinate systems in two frames, respectively. $x_{1}$ is the image point of p and $p^{\prime }$ in $O_{1}-xyz$. $x_{2}$ is the image point of p in $O_{2}-x^{\prime }y^{\prime }z^{\prime }$. $x_{2}^{\prime }$ is the image point of $p^{\prime }$ in $O_{2}-x^{\prime }y^{\prime }z^{\prime }$. $e_{1}$ and $e_{2}$ are the intersections of the baseline and the imaging planes. $l_{1}$ and $l_{2}$ are epipolar lines. R and T are the rotation and translation of the camera from $O_{1}-xyz$ to $O_{2}-x^{\prime }y^{\prime }z^{\prime }$, respectively. H is the homography matrix between the images of plane P in two camera coordinate systems, which maps $x_{1}$ to $x_{2}$.

Furthermore, a homography matrix can be calculated from $\vec{n_{1}}$ and $\vec{n_{2}}$. The homography matrix is usually used to describe the transformation between images when there is the same plane in two images. There is relative motion between the target and a camera in vibration. If we think the target position is the same, then the camera’s pose is different in two consecutive frames. For points out of plane, homography may not be appropriate for images taken in two camera poses. As Figure 6 shows, in the camera coordinate system $O_{1}-xyz$ and $O_{2}-x^{\prime }y^{\prime }z^{\prime }$ of two poses, $x_{1}$ and $x_{2}^{\prime }$ are the image points of $p^{\prime }$, which is a point out of plane P, and $x_{2}$ is the mapped point of $x_{1}$ using homography H. It is easily found that for a point $p^{\prime }$, the difference between $x_{2}$ and $x_{2}^{\prime }$ represents the error of homography. Additionally, we define the following: $O_{1}O_{2}$ is the baseline between two camera poses, $e_{1}$ and $e_{2}$ are the epipoles, and $l_{1}$ and $l_{2}$ are the epipolar lines. The difference between $O_{1}-xyz$ and $O_{2}-x^{\prime }y^{\prime }z^{\prime }$ is called the parallax. It should be noted that in the illustration herein, the two coordinate systems $O_{1}-xyz$ and $O_{2}-x^{\prime }y^{\prime }z^{\prime }$ represent different poses of the same camera, rather than two cameras in stereo vision, whose parallax far exceeds the description range of the homography matrix.

It is easy to prove that $\left|x_{2}^{\prime }x_{2}\right|$ depends on $\left|pp^{\prime }\right|$ and the norm of T (or length of $\left|O_{1}O_{2}\right|$ because $T=O_{1}O_{2}$). If the parallax between two images is low enough or there is only rotation of the camera pose between two images, in other words the translation |T| between $O_{1}-xyz$ and $O_{2}-x^{\prime }y^{\prime }z^{\prime }$ is small enough relative to the scene depth $\left|pp^{\prime }\right|$, the homography matrix will be accurate enough to describe the correspondence of all points in two images even when they are not on the same plane [17]. If the vibration-caused camera motion is small enough relative to the scene depth, the parallax between the two virtual frames is low and the homography matrix is sufficiently accurate for global pixel mapping. For virtual frame sequences that pass the frequency check, the corresponding feature points of the virtual frames can be extracted, and then the homography mapping between two virtual frames can be found.

Furthermore, as long as there are more than eight non-coplanar feature points in a pair, the accuracy of this correspondence can be evaluated by calculating $N_{L1}$ again after the corresponding points have been mapped by the homography matrix. In this sense, the method itself limits its scope of use, and excessive vibration or motion of the camera will be discovered during repeated L1-norm calculations to avoid meaningless or mistaken compensation. When the homography matrix is used to map one image to another, there is interpolation and pixel rounding in the process as digital images have integer pixel coordinates and gray values while feature points have sub-pixel level coordinates. By considering this, we introduced the average pixel displacement $d_{pixel}$ to indicate the consistency of feature points after the virtual frame was mapped by the homography matrix. This can be expressed as:

(11) $$d_{pixel}=\frac{\left|\sum_{I}\left(\vec{n_{1}}\left(I\right)-\vec{n_{2}}\left(I\right)\right)\right|}{N_{p}}$$

supposing that $\vec{n_{1}}=\left\{\left(x_{i},y_{i}\right)\right\}$, $\vec{n_{2}}=\left\{\left(u_{i},v_{i}\right)\right\}$, and $i=1,2,3,\ldots ,N_{p}$ is the order of feature points, we have $N_{L1}=\sum \sqrt{{\left(x_{i}-u_{i}\right)}^{2}+{\left(y_{i}-v_{i}\right)}^{2}}/N_{p}$ and $d_{pixel}=\sqrt{{\left(\sum x_{i}-\sum u_{i}\right)}^{2}+{\left(\sum y_{i}-\sum v_{i}\right)}^{2}}/N_{p}$, which means that $N_{L1}$ depends on the Euclidean distance between each pair of feature points, and $d_{pixel}$ depends on the Euclidean distance between the mean values of all feature points in two arrays.

**Figure 7:**
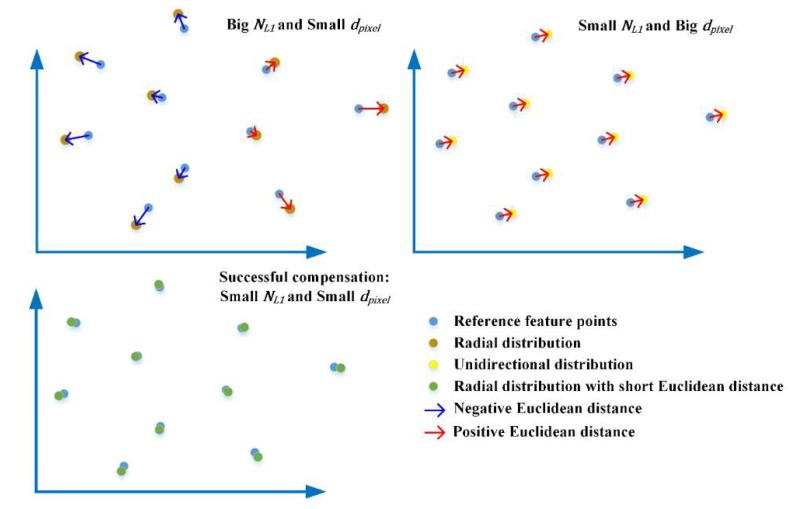
Difference between $N_{L1}$ (L1-norm) and $d_{pixel}$ (average pixel displacement).

The difference between $N_{L1}$ and $d_{pixel}$ is that $N_{L1}$ indicates the absolute difference between two point arrays, which is non-directional, but $d_{pixel}$ is directional, as shown in Figure 7. This means that if a point array is evenly radially distributed relative to another point array, it will have a small $d_{pixel}$ while the $N_{L1}$ is big. Conversely, if one set of points is unidirectionally distributed relative to the other, the $d_{pixel}$ may be big even when the $N_{L1}$ is small. The introduction of $d_{pixel}$ is to indicate the situation where after compensation, $N_{L1}$ decreases but the compensated virtual frame still has a unidirectional displacement to the reference virtual frame. According to the experiment in Section 2.1, the unidirectional displacement is critical to phase unwrapping.

**Figure 8:**
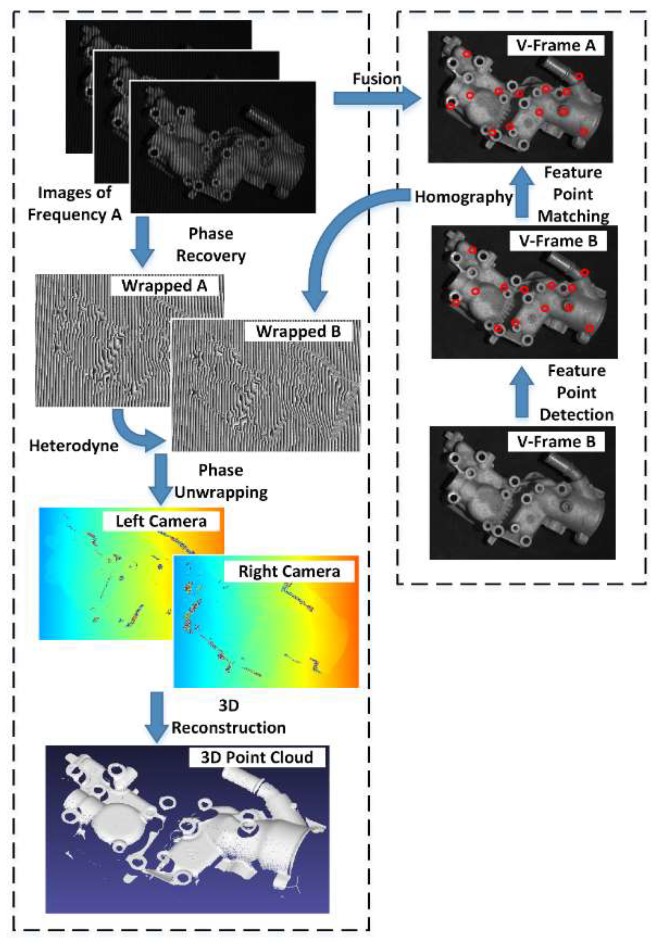
Block diagram illustrating the steps involved in traditional multi-frequency, phase-shifting methods (**left** column) and the proposed motion compensation algorithm (**right** column). Exemplary images are given for an experimental result at the output of each step.

In the multi-frequency, phase-shifting fringe projection methods, the wrapped phase is calculated using the images of the same frequency and the unwrapped phase is obtained using the heterodyne from different wrapped phase images [9]. From above, we know that the homography matrix can be used to map two images affected by vibration in a low parallax situation. If it is suitable for the homography matrix to map the virtual frames superimposed from the phase-shifting subsequence, it can also be applied to the wrapped phase maps. The heterodyne algorithm recovers the absolute phase information based on the phase values of the same pixel in different wrapped phase maps. Noises and errors of the unwrapped phase come from the destruction of the pixel correspondence. As the homography matrix obtained from the feature points is sufficiently accurate for the global pixel mapping in the vibration situation, it can be applied to correct the pixel correspondence between the wrapped phase maps. The operation flow is shown in Figure 8. For the sake of simplicity, we used the reconstruction process of a two-frequency, three-step phase-shifting as an example. Among the six images captured by the left camera, the three images belonging to the same frequency A can generate a wrapped phase map, Wrapped A. In our method, they can simultaneously synthesize a virtual frame, V-Frame A. Similarly, there are the Wrapped B and V-Frame B. Due to the influence of vibration, the pixel correspondence between Wrapped A and Wrapped B was destroyed. In our method, the SIFT (scale-invariant feature transform) method was used to detect feature points in V-Frame A and V-Frame B. The reason for choosing the SIFT method is that it is invariant in perspective transformation and is not sensitive to grayscale changes. Then two arrays of feature points were matched by the FLANN (fast library for approximate nearest neighbor) matcher and according to the average Euclidean distance, the mismatched pairs with excessive distance were screened out. Then, we obtained a homography matrix from two arrays of feature points that mapped V-Frame B to V-Frame A. We then applied the same homography matrix used to map Wrapped B to Wrapped A. Therefore, the pixel correspondence between them was corrected. Using the Wrapped A and corrected Wrapped B, we could obtain the unwrapped phase map of the left camera, where the same thing happens on the right camera. In the end, the correct 3D reconstruction results were generated.

## 3. Experimental Setup

It can be summarized that in this motion composition method, the features were detected on the superposition of phase-shifting fringe images and then the features of each frequency were matched to find the homography transform. The wrapped phase image of the second frequency was mapped by the homography to match the wrapped phase image of the first frequency. As mentioned in Section 2, this method has a limited scope of application, and works only when the homography mapping is sufficiently accurate for the global pixel correspondence, which mainly depends on the motion intensity between the frames and the effectiveness of the homography matrix. The low motion intensity guarantees the existence of a proper homography matrix, which can be indicated by our vibration detection method. Additionally, the feature point detection determines if the proper homography matrix can be accurately acquired. The main purpose of the experiments was to find out to what degree of motion the homography mapping was applicable and how to evaluate the validity of the calculated homography matrix.

**Figure 9:**
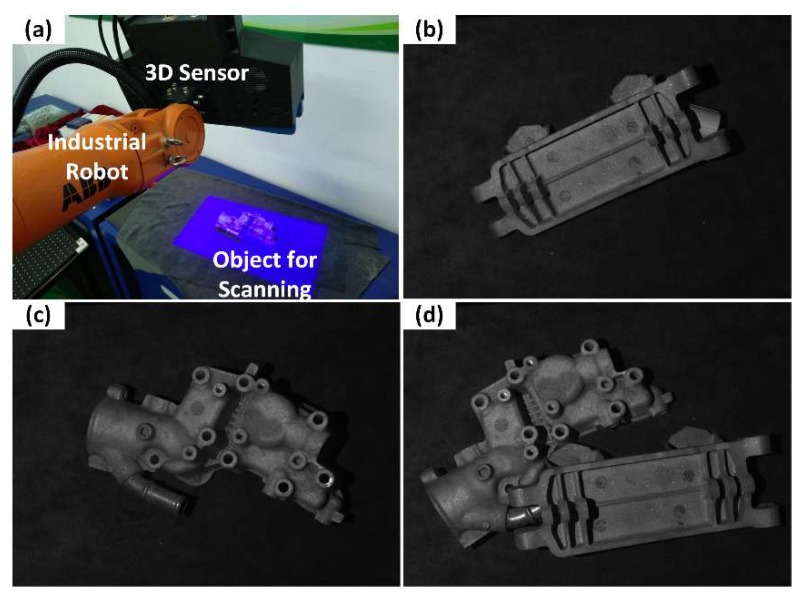
Experimental setup. (**a**) 3D sensor fixed to the end effector of an industrial robot. (**b**) Object 1 for scanning. (**c**) Object 2 for scanning. (**d**) Joint objects for scanning.

We applied the proposed method to a stereo vision 3D sensor that worked under the four-step, three-frequency phase-shifting principle. In order to simulate the vibration that causes linear motion, the sensor was fixed to the end-effector of an industrial robot. Figure 9a shows the experimental setup where we used the robot movement to simulate the vibration in the measurement process. It should be noted that the movement of the robot included both rotation and translation. To avoid the selectivity of the algorithm parameters to the scene, the scanned object was randomly selected each time from those in Figure 9b–d. The scene depth varied from 30 mm to 100 mm for different objects. In this simulation, we supposed that the vibration frequency was at most half of the sensor scanning frequency, which ensured that the scanning process took place in one-way motion.

## 4. Experiments and Results

**Figure 10:**
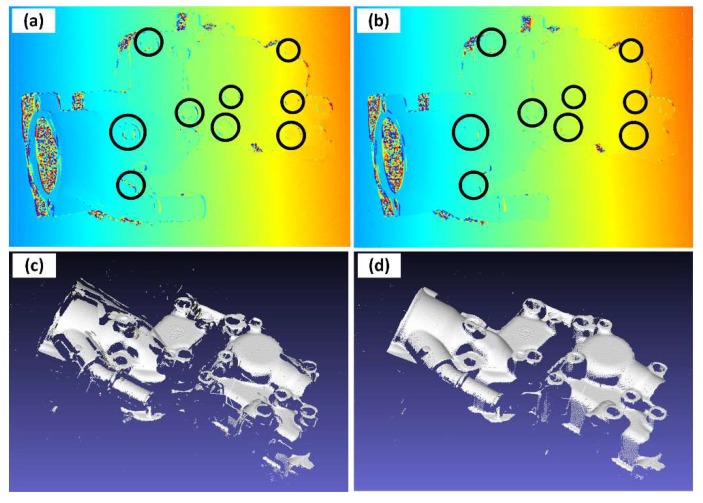
Phase unwrapping and 3D reconstruction in motion. Phase unwrapping result in motion with pseudo-color representation, where the same color represents the same phase and black circles emphasize easy-to-identify phase errors: (**a**) phase unwrapping result in motion, and (**b**) phase unwrapping result after compensation. (**c**) 3D reconstruction result in motion and (**d**) 3D reconstruction result after compensation.

An experiment on the 3D reconstruction from a multi-frequency phase-shifting sequence in motion was performed to validate the algorithms. Figure 10 shows the phase unwrapping and 3D reconstruction results in motion and after compensation. It is obvious that the phases at the bosses were severely damaged in Figure 10a (it should be noted that errors were not only at the bosses or edges but the phase errors at the bosses are most recognizable for human eyes). For the multi-frequency phase-shifting sequence, the main form of the motion error was “broken” surfaces, as shown in Figure 10c. After the compensation, the phase errors decreased greatly in the unwrapped phase image. As Figure 10b shows, the recognizable phase errors at the bosses (emphasized with black circles) were almost removed. The “broken” surfaces of 3D data in motion were also “repaired” after compensation in Figure 10d.

**Figure 11:**
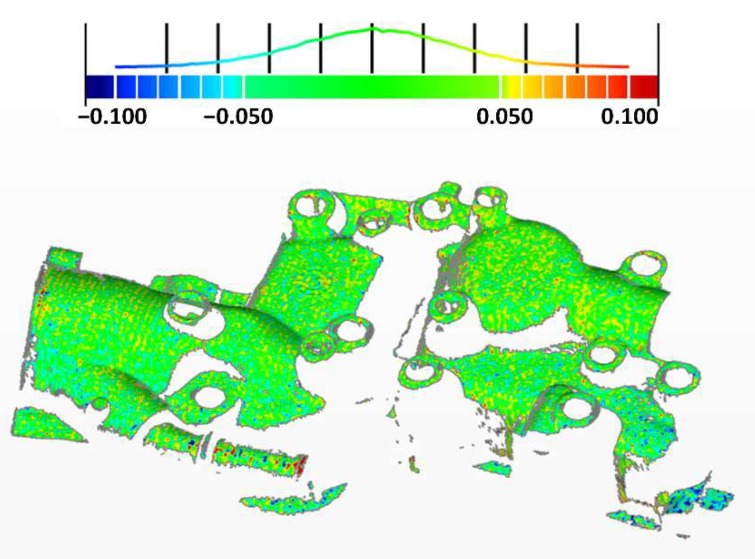
3D comparison of the static 3D data (reference) and the compensated 3D data (test) (unit: mm). The color represents the deviation value, and the green color indicates that it was within ±0.050 mm. Red indicates the positive deviation and blue indicates the negative deviation. The top curve represents the distribution of points with different deviations.

In order to evaluate the degree of conformity of the 3D reconstruction to the true value, we compared the compensated 3D data with the static 3D data, as shown in Figure 11. The average upper and lower deviations of the result were +0.055 mm and −0.054 mm, respectively. The deviation distribution curve is presented at the top of the figure, which obeys a normal distribution. Most of the overshoots were concentrated at the edges and high exposure areas. This may come from the alignment error because in this comparison, we transformed the reference 3D point cloud to closed surfaces composed of triangles, and then aligned and compared the test 3D point cloud with the closed surfaces. Still, most of the 3D reconstruction errors had been fixed.

In order to obtain the boundary conditions that limit the application of this method, we repeated the compensation and 3D reconstruction processes with different movement parameters (the motion speed percentage of the robot, which indicates the relative speed of motion respective to a fixed set value) and object placement. To avoid the introduction of prior knowledge, all criteria were derived only from the images themselves. Streak intensity and $N_{L1}$ were used to indicate the intensity of the vibration to find out what degree of vibration would exceed the compensation capacity of this method. The streak intensity has a priority to $N_{L1}$ because feature-point detection can only be applied to a virtual frame with a negligible fringe component. Even if the vibration intensity is within the compensation capacity of this method, there still needs to be enough credible feature points for the homography matrix calculation. In addition, the homography matrix needs to be sufficiently accurate to describe the correspondence between the feature point arrays from the two frames. If the above criteria are satisfied, $N_{L1}$ will significantly decrease after mapping.

In this experiment, we used the ratio of $N_{L1}$ after mapping and the original $N_{L1}$ to indicate the effectiveness of the homography matrix mapping, expressed as ”decreased $N_{L1}$”. Furthermore, we used the average pixel displacement $d_{pixel}$ after compensation to avoid the situation mentioned in Section 2.2 where $N_{L1}$ decreased, but the compensated virtual frame still had a unidirectional displacement to the reference virtual frame. It should be noted that the decreased $N_{L1}$ criteria have a priority over the $d_{pixel}$ criteria because even when mapped using a less accurate matrix, the frame may be partially corrected to achieve a better value of $d_{pixel}$ (as explained in Section 2.2). Grid sampling was adopted to avoid over-densification. As this is a stereo vision system, each value was the more representative one from the corresponding values of the two cameras.

**Figure 12:**
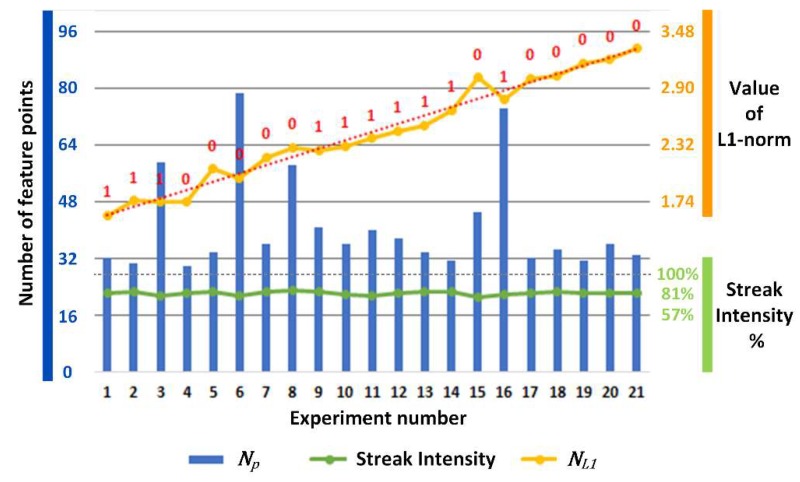
The number of feature points $N_{p}$, the streak intensity, and the $N_{L1}$ ordered by the motion speed percentage. The red dotted line shows a line fit curve for the $N_{L1}$. Data annotation “1” indicates successful compensation, and data annotation “0” represents the failed compensation.

Figure 12 shows the values of $N_{p}$, the streak intensity, and the $N_{L1}$ ordered by the motion speed percentage. $N_{p}$ is the number of feature points; the streak intensity is the ratio of the fringe frequency amplitude in the first virtual frame and the first fringe image in the sequence; and the $N_{L1}$ is the L1-norm of $\vec{n_{1}}$ and $\vec{n_{2}}$. In the data annotations, shown in red, state “1” indicates successful compensation, where the average deviation (average of absolute values of average upper and lower deviations to simplify the representation) of the compensated data and static data is below 0.095 mm (specific values can be found in the table at the end of this section), and state “0” represents the situation where there are still visible “broken” surfaces in the 3D reconstruction result after compensation.

For situations with an $N_{L1}$ over 3.210 (data number 20), the motion error was beyond the capacity of the compensation method, while the streak intensity was still below the threshold for feature point detection. From the results, we can conclude that the streak intensity was looser than $N_{L1}$ in indicating the vibration intensity. In addition, it was easily found that $N_{L1}$ was linearly related to the motion speed percentage, as the red dotted line shows.

**Figure 13:**
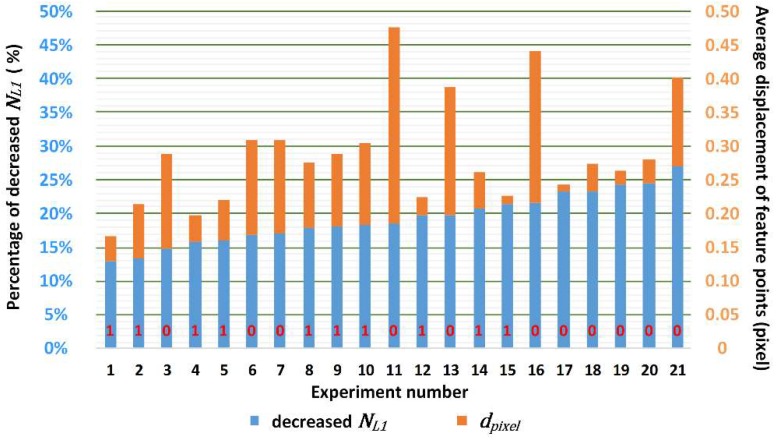
Compensation results in term of the decreased $N_{L1}$ and $d_{pixel}$, ordered by the decreased $N_{L1}$.

Figure 13 shows the value of the decreased $N_{L1}$ and $d_{pixel}$ for various experiments, ordered by the value of the decreased $N_{L1}$. The decreased $N_{L1}$ is the proportion of $N_{L1}$ after $\vec{n_{2}}$ is mapped by the homography matrix, compared to the original $N_{L1}$. The $d_{pixel}$ is the pixel displacement after V-frame B is mapped by the homography matrix. As shown in the figure, for situations where the decreased $N_{L1}$ is over 21.3% (cases 16–21), the homography matrix obtained from the feature points is not accurate enough for successful compensation. Furthermore, for an accurate decreased $N_{L1}$, if the $d_{pixel}$ is bigger than 0.121, the compensation will fail in the interpolation and pixel rounding process.

In the experiment, the thresholds of the $N_{L1}$, decreased $N_{L1}$, and $d_{pixel}$ were confirmed as 3.210, 21.3%, and 0.121, respectively. The original data supporting the graphs are listed in Table 1. The motion speed percentage and average deviation of each result can be referred from this table. It should be noted that the average deviation is the average of the absolute value of the 3D point cloud’s upper and lower deviations. $N_{L1}$ is a very good indicator for the vibration intensity. In fact, the approximate curve of the motion speed percentage and $N_{L1}$ had very good linearity if we considered the accurate value of the motion speed percentage. As we increased the motion speed percentage further, we found that until the streak intensity reached 90%, it was still possible to extract feature points while $N_{L1}$ was far beyond the boundary of this method.

Even now, the motion compensation algorithm still has a large optimization space in feature point detection, mismatch removal, robustness, and so on. We think that this is not the best performance of this algorithm. From the experiment, we can propose two bases for the compensation method to work.

(1) The vibration or motion intensity was relatively low for the scanning period, thus guaranteeing the existence of an accurate homography matrix.
(2) The features in the scene and the method for feature detection and matching ensure that a valid homography matrix could be accurately obtained.

Considering that grayscale feature point detection is not the only way to obtain the existing homography matrix, other methods of acquiring a homography matrix can be explored to extend the application space of the algorithm.

## 5. Conclusions

In this paper, we proposed a metric for the vibration intensity of a multi-frequency phase-shifting method and initially demonstrated the potential of the corresponding motion compensation method. The calculation of the metric is simple and straightforward, which makes it a very good indicator for the vibration intensity of the multi-frequency, phase-shifting sequence. Furthermore, this vibration detection method forms the basis of the motion compensation algorithm. The advantages of the compensation method are threefold. First, the compensation factor can be obtained directly from the image sequence without any manual operation or prior knowledge of the scene. Second, the isolation of pixels is retained in this method. Finally, all operations in the algorithm are global, which enables a fast processing speed. The 3D reconstruction of objects in the presence of vibration demonstrated the capability and good performance of the proposed method, rendering it a potential new technique for 3D measurement applications where vibration is inevitable.

The limitation of this work is that the compensation depends on feature point extraction, and matching and filtering algorithms, which limits the success rate of compensation. In our future work, we plan to improve the feature point extraction, matching and filtering algorithm, and try to optimize the homography matrix using phase information.

## Figures and Tables

**Table 1:** Original data of Figure 12 and Figure 13: Motion speed percentage, streak intensity, $N_{p}$, $N_{L1}$, decreased $N_{L1}$, $d_{pixel}$ and average deviation.

No.	Motion Speed Percentage	Streak Intensity	$N_{p}$	$N_{L1}$	Decreased $N_{L1}$	$d_{pixel}$	Average Deviation
1	14%	80.90%	40	1.607	21.30%	0.013	0.075 mm
2	15%	82.30%	38	1.757	12.90%	0.038	0.089 mm
3	15%	78.40%	74	1.744	17.80%	0.097	0.095 mm
4	16%	80.80%	37	1.747	17.10%	0.138	Failed
5	17%	82.90%	42	2.081	23.20%	0.010	Failed
6	17%	78.70%	98	1.992	23.20%	0.041	Failed
7	18%	81.80%	45	2.207	24.40%	0.035	Failed
8	19%	83.00%	73	2.304	19.80%	0.190	Failed
9	20%	82.30%	51	2.275	18.20%	0.107	0.067 mm
10	20%	79.00%	45	2.317	15.80%	0.039	0.065 mm
11	21%	78.20%	50	2.394	20.70%	0.054	0.091 mm
12	22%	80.60%	47	2.477	18.30%	0.121	0.055 mm
13	23%	82.50%	42	2.531	16.10%	0.060	0.061 mm
14	24%	82.00%	39	2.688	13.30%	0.080	0.079 mm
15	24%	76.60%	56	3.026	24.30%	0.100	Failed
16	24%	79.20%	93	2.795	19.70%	0.028	0.090 mm
17	25%	81.00%	40	3.014	27.00%	0.132	Failed
18	26%	81.70%	43	3.043	18.60%	0.290	Failed
19	27%	81.00%	39	3.170	24.20%	0.022	Failed
20	28%	81.00%	45	3.210	14.90%	0.140	Failed
21	29%	81.20%	41	3.318	17.10%	0.067	Failed

## References

[1] Han L., Cheng X., Li Z., Zhong K., Shi Y., Jiang H. (2018). A Robot-Driven 3D Shape Measurement System for Automatic Quality Inspection of Thermal Objects on a Forging Production Line. Sensors.

[2] Yao J., Chen X., Zhou Y., Miao H., Chen J. (2014). Phase error elimination considering gamma nonlinearity, system vibration, and noise for fringe projection profilometry. Opt. Eng..

[3] Huang H., Agafonov V., Yu H. (2013). Molecular electric transducers as motion sensors: A review. Sensors.

[4] Svinkin M.R. (2002). Predicting soil and structure vibrations from impact machines. J. Geotech. Geoenviron. Eng..

[5] Manap R.A., Shao L. (2015). Non-distortion-specific no-reference image quality assessment: A survey. Inf. Sci..

[6] Kim M., Yoon D.Y., Pahk H. (2015). Vibration Measurement Using a Fringe Pattern in Reflective Monochromatic Interferometry. J. Opt. Soc. Korea.

[7] Zuo C., Huang L., Zhang M., Chen Q., Asundi A. (2016). Temporal phase unwrapping algorithms for fringe projection profilometry: A comparative review. Opt. Lasers Eng..

[8] Li Z., Zhong K., Li Y.F., Zhou X., Shi Y. (2013). Multiview phase shifting: A full-resolution and high-speed 3D measurement framework for arbitrary shape dynamic objects. Opt. Lett..

[9] Reich C., Ritter R., Thesing J. (1997). White light heterodyne principle for 3D-measurement. Sensors, Sensor Systems, and Sensor Data Processing.

[10] Zhang S. (2009). Digital multiple wavelength phase shifting algorithm. Optical Inspection and Metrology for Non-Optics Industries.

[11] Weise T., Leibe B., Van Gool L. Fast 3d scanning with automatic motion compensation. Proceedings of the 2007 IEEE Conference on Computer Vision and Pattern Recognition.

[12] Cong P., Xiong Z., Zhang Y., Zhao S., Wu F. (2015). Accurate dynamic 3d sensing with fourier-assisted phase shifting. IEEE J. Sel. Top. Signal Process..

[13] Lu L., Xi J., Yu Y., Guo Q. (2014). Improving the accuracy performance of phase-shifting profilometry for the measurement of objects in motion. Opt. Lett..

[14] Feng S., Zuo C., Tao T., Hu Y., Zhang M., Chen Q., Gu G. (2018). Robust dynamic 3-D measurements with motion-compensated phase-shifting profilometry. Opt. Lasers Eng..

[15] Li Z., Shi Y., Wang C., Wang Y. (2008). Accurate calibration method for a structured light system. Opt. Eng..

[16] Zuo C., Chen Q., Gu G., Feng S., Feng F., Li R., Shen G. (2013). High-speed three-dimensional shape measurement for dynamic scenes using bi-frequency tripolar pulse-width-modulation fringe projection. Opt. Lasers Eng..

[17] Hartley R., Zisserman A. (2003). Multiple View Geometry in Computer Vision.

